# On Wounds of the Scalp, and Their Consequences

**Published:** 1831-01-01

**Authors:** 


### ST. GEORGE'S HOSPITAL.

### On Wounds of the Scalp and their Consequences.

In our last report from this hospital we took up the consideration of injuries of the head, and detailed some interesting cases of concussion and its consequences. We propose to follow up the subject, by relating some of the cases of scalp-wound which have lately been treated at the hospital.

If an individual receives a wound of the scalp which merely divides the superficial soft parts, and does not penetrate the tendon of the occipito-frontalis, no unpleasant consequences usually follow, but it heals nearly as a wound elsewhere. We are supposing that the symptoms of concussion are slight, and do not materially interfere with the favourable progress of the case. There is no reason why a scalp-wound should be unaccompanied by very severe symptoms of concussion or compression, but that is not the case which we are now considering. If the wound has extended through the tendon of the occipito-frontalis and laid bare the bone, the injury is universally allowed to be more severe. In the first place the violence has probably been greater, and there is consequently more danger to be apprehended from injury to the parts within the skull; and in the second place, such a wound is more likely to be followed by common erysipelas, or a peculiar variety to which we shall presently advert. The bone which is laid bare may die, and exfoliate; or the dura mater beneath may be separated from it, and matter may form between them. All these are dangers which the voice of the profession has attributed to such wounds as we have mentioned. Scalp-wounds are also dangerous when very extensive, and complicated with much effusion of blood, or the diffusion of foreign bodies, as gravel and dirt, between the scalp and the occipito-frontalis. Such wounds very rarely escape an attack of erysipelas in this hospital.

Perhaps a simple and yet comprehensive arrangement of scalp-wounds may be made in the following way. They may be conveniently divided into four varieties, differing very much in the effects which they usually produce.

1mo. Such wounds as are not very extensive nor much contused and lacerated, and in which the occipito-frontalis is not torn, nor the bone denuded.

2do. Those wounds which are not very extensive, but in which a certain portion of the bone *is* denuded.

3tio. Those wounds which *are* very extensive; with contusion, laceration, and their consequences: the occipito-frontalis torn: and the bone more or less extensively denuded.

4to. Wounds which may or may not be extensive, but are complicated with fracture of the skull, constituting "compound fracture of the cranium."

Of these the first are the least dangerous, the last the most so. Of the former we need give no examples, as most individuals have witnessed many, and the progress to a cure is seldom disturbed except by fortuitous circumstances, or a casual attack of erysipelas. On "compound fracture of the cranium" we shall say nothing, as it deserves and will obtain a separate consideration hereafter. The varieties of scalp-wound to which we direct our readers' attention, are the second and the third upon our list.*

And first, of those wounds which are not very extensive, but in which a certain portion of the bone is denuded. These have always been looked on with suspicion, and are generally watched with care. The shock and disturbance occasioned by the accident having passed away, there is usually a quiescent period, which may either end in the healing of the wound, or in more unpleasant consequences. The descriptions of some authors would lead their less experienced readers to believe, that if a portion of the cranium is exposed, it must necessarily die and be cast off by the process of exfoliation. Cases in proof of the contrary are ocring daily. We may briefly mention the particulars of one or two.

CASE 1. *Slight Scalp-wound—Bone denuded—cure.*

John Reed, aged 25, a sturdy-looking bricklayer, was admitted on the 15th of June last, under the care of Mr. Babington. There was a small irregular scalp-wound at the vertex of the head, without much surrounding tumefaction. The bone was denuded for a small extent. There were fulness of pulse and heat of skin.

The accident had occurred on the preceding afternoon, from a brick falling on his head with such violence as to be itself split into three pieces. He was slightly stunned at the time. The treatment consisted of two bleedings and moderate purging. On the eighth day the wound was healed, and in two or three days more the man was discharged cured.

CASE 2. *Slight Scalp-wound—Bone denuded—Symptoms of Compression—Cure.*

James Patterson, ætatis 60, admitted July 16, 1830, under the care of Mr. Keate.

Wound of the scalp on the crown of the head, leading down to the bone, which is denuded for the extent of a half-crown piece. Sensibility not quite perfect. Besides this, he has several other injuries, particularly an ugly lacerated wound of the lower lip. A short time before his admission he had been kicked in the mouth by a horse, and the scalp-wound appeared to have been inflicted by the violence with which the occiput struck the ground. He had been insensible for some time, but had not vomited.

The scalp-wound was dressed with adhesive plaster, and the other injuries properly attended to. In the course of two hours the pulse became full, sharp, 96—he was less sensible—there was a kind of stertor—and there were some convulsive twitchings of the lower limbs.

*V. S. ad* ℥xij.

The bleeding afforded much relief, and the blood was buffed and cupped. Next day he had again relapsed into some degree of stupor—the pulse was 80, full—the skin hot—the pupils contracted.

He was bled a second time, and had antimoniated salines. He was bled again on the 18th. The head symptoms had already subsided, and the general excitement now passed away—the scalp wound gradually healed, and no scale of bone was thrown off—and on the 11th of August the patient was dismissed from the hospital cured.

The preceding case was chiefly remarkable for the rapid manner in which reaction was established, and the early buff upon the blood. It is probable from these circumstances, as well as from the facility with which the head

* As the symptoms of concussion or compression may readily be combined with all kinds of scalp-wound, but are not essential accompaniments of any, we discard the consideration of them at present altogether.

symptoms disappeared, that the latter were rather occasioned by the inflammatory action set up in the brain than by extravasation. The two cases prove that portions of the skull may be denuded of pericranium and exposed to the influence of the atmospheric air, without any exfoliation ensuing in consequence.

But these accidents may give rise to common erysipelas of the scalp, or to a peculiar affection, allied to erysipelas and running into it, and having its seat in the cellular membrane beneath the tendon of the occipito-frontalis muscle. And first of erysipelas of the scalp.

CASE 3. *Scalp Wound—Bone exposed—Erysipelas—Active Treatment—Recovery.*

Anthony Hitchcock, a soldier, ætatis 30, admitted Nov. 24th, 1827, under the care of Mr. Jeffreys.

Has a jagged wound, of almost sloughy appearance, on the left side of the forehead, with the bone slightly exposed. Another contusion behind the ear. Much pain in the head—eyes suffused and heavy—pulse strong and labouring—tongue furred—bowels confined.

States that four days ago he fell from a coach, was picked up insensible, and was carried to some hospital. Here he remained for a day or two, but on recovering his sensibility he left the hospital, and went to the public-house where he was billeted. He lay in bed, without medical advice or assistance, till the time of his admission into this institution.

*Wounds dressed. V. S. ad* ℥xx.—*Calomel,* gr. ij. *Pulv. jalapæ,* gr. vj. 4*tis horis.*

He was relieved by the bleeding, but the bowels not having been opened, he took the senna draught every three hours until they were so. On the 25th there was still some pain in the head—the tongue was furred—the pulse rather more full.

*Cat. lini capiti—V. S. ad* ℥x. *H. salin. c. mag. sulph.* ʒss. 6*tis horis. P. c. calomel.*

The bleeding produced slight faintness, and in the evening he had some shivering; he had felt the same on the preceding day. On the 26th he was suffering much from head-ache and pain in the forehead—pupils rather dilated, with intolerantia lucis—pulse frequent and full—tongue whitey-brown and thickly coated—thirst—anxiety—pain just under the left nipple, where, on examination, a rib was found to be fractured. The blood drawn on the 25th was buffed and cupped.

*Bandage to the chest. Lot. spt. fronti. V. S. ad* ℥xv.

On the 27th the erysipelas had rather extended, with head-ache, thirst, furred tongue, pulse 92 and hard; the bowels were much purged. He was ordered to be bled to 20 ounces. In the evening he was not relieved, though the hardness of the pulse had disappeared; the pupils were contracted and sluggish; the patient inclined to be delirious. He passed a restless night, and on the next morning the erysipelas had extended down the right cheek; there was less pain in the head, the right side of which was œdematous; the tongue cleaner; pulse frequent and full; constant nausea. The blood drawn on the preceding day was a little cupped, but instead of the common buff, it shewed upon its surface a thick layer of perfectly translucent albumen, resembling jelly. The clot was loose. The bleeding was ordered to be repeated to 20 ounces, but 30 were taken by mistake; this blood shewed no trace of inflammation. The erysipelas now began to fade, the pains in the head and symptoms of pyrexia passed away, a state of considerable weakness ensued, but the wounds healed favourably, convalescence followed, and on the 9th of January the patient was discharged from the hospital cured.

CASE 4. *Scalp-wound denuding the Pericranium—Erysipelas—Recovery.**

W. Kelly, æt. 36, admitted Feb. 20th, 1829, under the care of Mr. Keate.

* As the bone itself was not denuded, it is hardly consistent to introduce the case in this place. The tendon of the occipito-frontalis, however, was torn, which makes the injury more severe than a superficial scalp-wound.

On the 17th he had received a small scalp-wound on the posterior part of the side of the head, by which the pericranium was denuded; there were no symptoms of concussion. The wound was dressed, and he resumed his work, but a rigor ensued, and he applied at the hospital with urgent symptoms of pyrexia on the 19th. When admitted, on the 20th, he complained of much pain in the head, the pulse was full and hard, the tongue white and coated. The lips of the wound were asunder. He was bled to 10 ounces, had calomel and house-physic, and was ordered salines with antimony and magnes. sulph. every six hours. In the evening the bleeding was repeated, and the blood drawn on both occasions was highly buffed and cupped.

On the 21st, an erysipelatous redness appeared upon the scalp; the pulse was 112, the skin hot, the tongue white. He was ordered the draught of the acetate of ammonia.

The erysipelas extended over the forehead, accompanied with an œdematous condition of the scalp, particularly near the wound. On the 23d, the pulse being frequent and not so full, the skin tolerably cool, the tongue moist, the bowels rather purged, and the erysipelas extending over the face, he was ordered the liq. amon. acet. with mist. camph. and tinct. op. ♏. x. 6tis horis. On the 24th the face was much swollen and of dull red colour, the tongue brownish in the centre, the pulse frequent without force, the manner occasionally incoherent. He was put on decoction of bark and the liq. ammon. acet.

On the next day the erysipelas had ended in desquamation of the cuticle, but still much swelling and a brownish red colour of the forehead, face, and neck remained. The puffiness and œdema about the side of the head were diminished. The pulse was lower, and the tongue more brown and dry. He was still light-headed and exceedingly restless and fidgetty, but would answer questions rationally enough. The parts had hitherto been treated with cold spirituous lotion, but now the erysipelas ointment spread on a linen mask was applied to them.* From this time the improvement was progressive, and convalescence was scarcely retarded by the formation of one or two subcutaneous abscesses, which were opened with the lancet. In the course of a short time the patient was discharged cured.

CASE 5. *Scalp-wound—Bone denuded—Erysipelas—Recovery.*

George Foley, a labourer, ætat. 32, admitted April 28th, 1829, under the care of Mr. Keate.

On the right side of the head, a little above and anterior to the ear, a jagged scalp-wound, leading down to exposed pericranium, and bare bone. The patient was not perfectly sensible, unable to answer questions, restless, and fidgetty; the pupils sluggish; the pulse but little affected. The portio dura on the same side appeared to be slightly paralyzed. There was a trifling injury about the knee-joint. The injury had been inflicted by a fall from a scaffold 40 feet in height; he had been completely stunned at the time, but had not vomited. The wounds were dressed, and cold lotion applied.

In the evening the pulse rose, and he was bled to 10 or 12 ounces; had a purge; and took salines with sulphate of magnesia every four hours.

On the next day he was so far recovered as to give a very clear account of the manner in which the accident had happened; there was more pulse, and the pupils were a little contracted; the paralysis of the portio dura, if such there was, had diminished. In the afternoon he became flushed, and the pulse had more force; in the evening he was bled to 10 or 12 ounces.

30th. Drowsy and heavy—pupils rather more contracted—pain in the site of the wound, which looks foul, and has been poulticed—pulse full, but not hard—skin warm—no rigor nor sickness. Blood drawn last night is not inflamed. Antimony was added to the

* The erysipelas ointment consists of equal parts of cerat. calaminæ, ceratum saponis, and unguentum plumbi acetatis.

salines. On the 1st of May there was less pain, but the pulse had some fulness and jerk, and the man continued heavy and drowsy.

*V.S. vespere ad* ℥x.

He passed a bad night, and on the 2d, all the symptoms were more severe. More head-ache—pulse frequent and bounding—skin hot—scalp tender about the wound and tumid. He had experienced a kind of chill, not amounting to a rigor. He was bled to ℥viij. and soon afterwards the blood shewed the buffy coat. In two or three hours another vein was opened, and 30 ounces of blood abstracted. This had a marked effect upon the pulse, the skin grew pale, and perspiration succeeded. At 5, p. m. reaction had not set in, the pulse was soft, the patient in a tranquil state.

Next day an erysipelatous blush had shewn itself upon the forehead, attended with tumefaction of the scalp. The skin was moist, the pulse soft, the tongue whitish, the head-ache trifling. He was ordered effervescing saline draughts. On the 4th, the erysipelas had extended down the forehead to the face, and the tint was very pale. The pulse was soft, and the other symptoms as favourable as could be wished. On the 5th the patient felt low, and bark was exhibited, in combination with the liq. ammon. acet: Afterwards the bark was given alone, a generous diet was allowed, convalescence made speedy progress, and in the course of a fortnight or three weeks the patient was discharged perfectly well.

These cases appear to us particularly interesting, and calculated to give rise to reflections of much consequence in a practical point of view. The symptoms in all the three were those of inflammatory fever, indicating the active treatment which they received. These are almost the only instances of erysipelas at this hospital, in which we have seen venesection performed, for the usual run of cases are as different from these as if they had a separate place in the nosology. We have said that the symptoms were inflammatory; we might have gone further, and asserted that they were not to be distinguished from phrenitis. Indeed all the cases were treated for inflammation of the brain, until the appearance of the erysipelatous blush determined the nature of the affection. On other occasions we have seen the same mistake occur, and eminent surgeons have directed active antiphlogistic measures, for supposed inflammation of the brain, when the symptoms have turned out to be those of erysipelas. Of course we are speaking of injuries of the head.

Practically speaking, the diagnostic error is of little importance; for, if the symptoms ushering in traumatic erysipelas ape phrenitis so closely, as scarcely to admit of discrimination, it is probable that the same, or nearly the same treatment is required, as if that affection were actually present. Nay, we cannot affirm that phrenitis does not precede the erysipelas, and that active measures are not necessary to arrest it. From these considerations we may conclude that it is scarcely possible to pronounce on the exact nature of the symptoms, until the appearance of the erysipelatous blush has determined the question; and athough we felt confident of the approach of erysipelas, we ought still to use active antiphlogistic measures, provided the symptoms clearly indicated their employment. At the same time, the surgeon should remember that what he sees may end in erysipelas only, and that this will not be benefited by such violent depletion as phrenitis would require. He ought also to be upon the watch lest the blush should escape his observation. We think that in most of these cases some œdema of the scalp precedes the redness, and may commonly be looked on as the herald of its approach.

The pyrexia preceding and accompanying erysipelas of the scalp, usually take place at a period anterior to the symptoms denoting the formation of pus upon the dura mater. There is also less rigor and more fever in the former than the latter. Rigors and sweating, with little heat of skin and excitement, characterise the presence of pus as under the dura mater; whilst much excitement and heat of skin, with slight chills or moderate rigors, usher in the attack of erysipelas. This is a point deserving attention in other cases than those immediately under consideration. Generally the symptoms of erysipelas occur at an earlier period than those of the formation of pus within the cranium, or of the purulent depôts in the lungs and liver. In the first case which we have here recorded the accident occurred on the 20th November, and the erysipelas *appeared* on the 26th —in the second case, it appeared on the fourth day after the infliction of the injury—in the third, upon the fifth day. The preliminary symptoms of fever commenced in all within two days from the occurrence of the accident. These are obvious distinctions between the two affections, even prior to the development of the erysipelatous blush.

The state of the wound requires a remark. It assumes an unhealthy appearance, but not an alarming one. During the existence of the fever and erysipelas it makes no advance to cicatrization, but the bone does not die, and, after the subsidence of the erysipelas, the healing process generally proceeds with more rapidity.

We hope these observations on traumatic erysipelas of the scalp will not be considered out of place. They have nothing to recommend them except their being founded on attentive observation of cases. In conclusion we may simply remark, that after scalp-wounds, and more especially after those in which the tendon of the occipito-frontalis is torn or the bone denuded, a series of symptoms ensue in the course of a few days, closely resembling those of phrenitis, or actually produced by it—and that, whether in consequence of the active treatment employed, or the efforts of the vis medicatrix naturæ, these symptoms end in the appearance of erysipelas of the scalp. For this variety of erysipelas incisions are seldom required. So much for common erysipelas of the scalp.

It has long been observed that scalp-wounds are occasionally followed by a very dangerous affection, differing in many respects from the ordinary character of erysipelas. Surgeons have attributed these severe accidents to wounds of the aponeurosis of the occipito-frontalis muscle, and the inflammation induced in that tendinous structure. Many hints on this subject will be found in the older writers on surgery, but, not to trouble our readers with quotations, it is sufficient to state that such were their opinions and ideas. One of the latest practical authors, Sir Astley Cooper, has made the same statement in his published lectures, and informs us that inflammation of the tendon of the occipito-frontalis is not uncommon, and abscesses, requiring incisions, are its frequent consequences. The matter, says Sir Astley, if the constitution has strength to produce it, is formed between the tendon and the pericranium—it cannot find its way out unless incisions are made through the tendon, and the inflammation accompanying the process sometimes destroys life. Sir Astley likewise observes that the inflammation of the tendon extends over the whole surface of the head, covering the scalp and face, and assuming an erysipelatous character, although it is not true erysipelas. It has not the vesicles usually attendant on the latter. Mr. Samuel Cooper, whilst reviewing in his Surgical Dictionary the opinions of Mr. Pott on this subject, opinions which, though imperfect, were yet remarkably sound and correct, contrasts them with those of M. Dessault, and decides in favour of the latter. Mr. Pott had observed that incisions were frequently necessary, M. Dessault decided that they were not, and Mr. Cooper cleaves to Dessault and renounces Pott. Unfortunately this is not the only instance in which Mr. Cooper weighs two opinions in the balance, and prefers the wrong one.

Prior to entering on the symptoms of this particular affection we should briefly advert to the structure of the parts which it attacks. The cutis of the scalp is particularly firm and dense —the adipose tissue firm—the cellular membrane connecting it to the epicranium or tendon of the occipito-frontalis, neither distinct nor readily admitting of effusion into its cells. The structure of the tendon of the occipito-frontalis need scarcely be alluded to here; suffice it to say that, like all aponeuroses, its texture is firm and unyielding. Beneath the tendon of the occipito-frontalis is a very lax cellular membrane, connecting it loosely with the pericranium. The contrast between the cellular tissue above and below the occipito-frontalis is very marked. Above it, serous infiltration is produced with difficulty, and never attains considerable magnitude; below, effusion takes place with facility, spreads to a great extent, and is only confined by the unyielding tendon of the occipito-frontalis. The laxity of this cellular tissue is obvious in the common operation of opening the cranium. On dividing the scalp through the tendon, it readily tears from the pericranium, but no one could separate the superficial parts of the scalp from the tendon without considerable force, if even he succeeded in effecting it at all.

Surgeons have attributed the extensive suppurations and sloughings that sometimes follow wounds, to inflammation of fasciæ or tendons. There can be little doubt that in this they were usually mistaken. The seat of the mischief has been the loose cellular membrane beneath the fascia or aponeurosis, and not the fascia itself. Inflammation takes place in this situation; the cellular membrane is loaded with effusion, which is bound down by the strong fascia; much compression is exercised in consequence on the cellular tissue, which possesses but a low degree of vitality; the inflammation partly ends in suppuration and the effusion of lymph, partly in sloughing, aided no doubt in the pressure exercised on one hand by the effusion, on the other by the fascia. The latter does not so readily slough, and perhaps the whole cellular texture beneath may be killed, before any natural process would form an opening for the exit of the matter pent up. Then, indeed, we find the fascia dead and sloughing, but this was not the original ill, it was the consequence of the suppuration and sloughing of the cellular membrane. This is a subject which cannot be treated in a few lines; it would require pages. But what we have said, was necessary, in order that the affection we shall now proceed to describe may be better understood.

A man receives a contused scalp-wound, which may or may not penetrate the tendon of the occipito-frontalis, but usually in this affection it does so. In the course of a few days the wound looks foul, its edges and immediate neighbourhood are puffy, and there is some firm œdema of the scalp around. The œdema and swelling of the scalp extend until the whole is extremely enlarged, very firm, and pitting only on steady pressure, whilst the indentation left by the finger is not removed so soon as in common œdematous swellings. If the head is shaved a slight erythematous or tawny blush may be seen here and there upon the scalp, but it has not the distinctive characters of erysipelas, nor is any disposition shewn to vesications. In some cases there is no perceptible discolouration whatever. Sometimes on the forehead, the back of the neck, or on the integument any where skirting the scalp, a dull erysipelatous blush may be observed.

If the progress of the disease be not arrested by nature or art, the swelling extends to the ears, the forehead, face, and neck, nay, even down to the chest or back. Where the common integuments are affected there is more of the true erysipelatous blush, but it is never very florid, the parts are much swollen, and the œdema remarkably firm. At this time the patient usually dies with low delirium, prostration, and coma. In the commencement of the affection there are often symptoms of considerable excitement, but so soon as it has reached the extent we have portrayed, and generally long before that period, the prostration of the powers of life has become very marked.

Let us now describe what may be called the anatomical characters of the disease. If a deep incision is made through the scalp in the early stage, where there is only swelling and firm œdema, the following is the condition of the parts. The cutis and adeps are nearly in a natural state, and the former bleeds freely. In the cellular membrane between the adeps and the tendon of the occipito-frontalis, there is seen a thin stratum of translucent gelatinous effusion, consisting more of lymph than serum, and filling all the cells. The tendon itself is little if at all altered in appearance. It is separated to the distance of a quarter of an inch, nay more than that, from the pericranium, by the same sort of gelatinous effusion in the loose cellular membrane, which was seen in a much less degree above the tendon. When cut, these parts suffer no collapse, although a quantity of serous fluid escapes. Neither the cellular membrane, in which the effusion is situated, nor the tendon bleed when divided, at least they commonly do not. Such is the state of the parts in the early stage.

If the incision be made at a more advanced period, the translucent gelatinous effusion is replaced by one more opake, and instead of serum, pus, or pus mixed with serum, will escape. The tendon is now stretched to the utmost, and appears to be losing its vitality, and in the cellular membrane below it sloughy shreds are mingled with the pus and lymph. The cellular membrane above the tendon shares the same fate with that below, but it is much less inclined to slough, and the disorganization has not proceeded so far. If the affection still hold on its course and the patient dies, the whole cellular membrane beneath the occipito-frontalis is converted into a mixture of pus and slough—the scalp when divided falls off the pericranium — the tendon itself is in many places sloughy —the pericranium is easily separable from the bone, which in parts is actually laid bare—and the whole presents such a mass of mischief as Mr. Pott only could picture with effect.

From the foregoing description we may come to the conclusion that the first material change in the organization of the parts of the scalp, consists in an effusion of gelatinous-like lymph and serum in the cellular membrane beneath the occipito-frontalis; and if that effusion be allowed to proceed, that extensive suppuration and sloughing will ensue. The masculine mind and practical experience of Mr. Pott induced him to practise and recommend incisions, and although that method of treatment has been impugned, on what we must call theoretical grounds, yet many experienced surgeons have continued to employ it with more or less decision. Sir Astley Cooper recommends incisions in order to let the matter escape, but he does not appear to have recourse to them previous to the occurrence of suppuration. Mr. Brodie, to whose excellent clinical instructions we are indebted for the delineation which we have given of the disease, is, as far as we know, the only surgeon who practises incisions to prevent the formation of pus, rather than to let it out when formed. Mr. Brodie makes free scarifications in the early stage, when only gelatinous effusion is found beneath the occipito-frontalis. The effect is in many cases most satisfactory, the extension of sloughing and suppuration being moderated when it is not entirely prevented. As the affection is of a highly dangerous character, it cannot be supposed that even scarification will always succeed, but we may safely affirm that it does so more frequently than any other plan of treatment. In fact, if the case be at all severe, the patient is inevitably lost unless incisions are promptly and boldly made. The following case will exhibit the efficacy of this plan of treatment.

Case 6. *Scalp-wound denuding the Tendon of the Occipito-frontalis—Affection of the Cellular Membrane beneath it—Incisions—Recovery.*

Hamlet Kemp, a labourer, ætatis 51, admitted Sept. 21, 1830, under the care of Mr. Brodie.

Lacerated scalp-wound, three inches in length, at the back part and right side of the head, exposing the tendon of the occipito-frontalis but not passing through it—much effusion into the surrounding soft parts. He received the wound two hours ago by falling from a bank at the river's side into a barge. He was not stunned. He has probably lived intemperately.

*Cataplasma lini.*

Next morning he had pyrexia, felt sick, and said he had had a rigor. The lips of the wound were rather tumid and inflamed, and firm tumefaction, with slight œdema, were observed around it.

*H. Sal.* c. *Vin. Ant. t.* ℳ. xx. 6*tis. hor.*

On the 23d the firm tumefaction round the wound was increased, and there was œdema over a great part of the scalp. The pulse was frequent and rather low, the tongue white and pasty. Mr. Brodie made free incisions in the scalp, down through the tendon of the occipito-frontalis. There was much gelatinous deposition in the cellular membrane beneath the tendon of the occipito-frontalis—some, but much less effusion of the same kind, between the tendon and the adeps. The effusion altogether was very extensive, the scalp being half an inch or more in thickness. He was ordered a poultice, and put upon decoction of bark, as the erysipelas prevailing in the hospital had universally assumed a very low type.

On the 24th, the œdema had extended but little on the back of the head—there was no distinct redness of the scalp. The appearance of the cellular membrane under the occipito-frontalis had changed from that of gelatinous lymph to a more purulent deposite. His manner was somewhat hurried, and he felt rather sick. In the evening he was rather delirious and passed a restless night. Pale granulations had appeared next morning above the tendon in some of the incisions—rather more thin discharge—no redness of the scalp. He had taken a pint of wine daily; it was now diminished to half a pint. Some bleeding took place in the night from one of the incisions. On the 26th the wounds were looking better, and a free secretion of pus was established in the cellular membrane above and below the tendon. On pressing the tumefied scalp pus issued from the incisions. The wounds were now syringed well with the solution of the chloride of soda; he had a mutton-chop daily.

Some diarrhœa now took place and was checked by the usual means. Healthy granulations sprang up from the sides of all the incisions and from the pericranium—considerable sloughs of the cellular membrane were drawn away—a great deal of pus which had formed, obtained a free discharge—and nothing occurred to retard the steady progress towards a cure. It has been mentioned that the original wound extended to the tendon of the occipito-frontalis, but not through it. When the incisions were employed, this portion of the tendon was not divided. The consequence was that it sloughed, and so did the pericranium below it, leaving the bone denuded for a space as large as a half-crown piece. This space was hemmed in and contracted by granulations—red vascular points appeared in the outer table, and at present, Oct. 21, there is every prospect of the scale of bone that is to separate being very inconsiderable. The wounds are nearly healed, the patient's health quite re-established.

CASE 7. *Scalp-wound denuding the Bone—Affection of the Cellular Membrane beneath the Occipito-frontalis—Incisions—Death.*

Mary Connor, ætatis 42, single, and a servant, admitted Sept. 15th, 1830, under Mr. Brodie.

Scalp-wound on the upper and back part of the head, situated transversely, two inches in length, denuding the bone, which feels rough. No loss of sensibility—surface cold — pulse slow. Fell from a pair of steps, twenty minutes ago, and struck her head against some flower-pots. Was stunned for some minutes, recovered, and vomited.

The wound was dressed.

At 3, p. m. she vomited, and soon afterwards there was some re-action, and pain in the loins. She was bled to ten ozs. and took salines with antimony. Next day there was much pain in the head, thirst, pulse 96, occasionally intermittent, and the bowels were not opened. The blood was not inflamed. She was bled again to six ozs., had senna, and a poultice to the wound. On the 17th the wound looked dry, there were excessive tenderness and slight œdematous swelling in its vicinity. The pulse was 108, full, and irritable — tongue white and moist — she had an anxious look.

*Hirud.* xv. *nuchæ*—*Cal.* gr. iv.—*Ext. Col. c.* gr. x. *stat.*

In the evening the scalp was more œdematous, and a faint, pale, erysipelatous blush had appeared upon the forehead, at the root of the hair. On the 18th, the erysipelas had extended over the forehead, and a firm œdema was spreading over the scalp. The edges of the wound were rather foul and yellowish. The patient was extremely nervous and depressed, and had been violently sick in the morning—pulse frequent but not full—no distinct pain *in* the head. Spirit lotion was applied—she took salines with the liq. op. sed. and had arrow-root allowed her.

On the 19th the erysipelas was extending over the face, with some tumefaction, and a muddy pale tint—there was no redness on the scalp. The symptoms were low, and there was the most remarkable debility of the voluntary muscles. The sp. ammon. arom. was added to the medicine. On the 20th the erysipelas was extending down the face with much firm tumefaction—there were vesications on the palpebræ. There were no head symptoms, but she had vomited. In the evening she was attacked with rather violent delirium, but did not evince any disposition to injure herself or others. The delirium continued on the 21st, the erysipelas had rather extended, the tumefaction of the face was considerable, firm, and painful. The scalp was firm and œdematous, but puffy in the immediate neighbourhood of the wound. She had felt chilly in the night. Incisions were made freely. In the occipital region a mixture of pus and lymph was found in the cellular membrane above and below the occipito-frontalis, but chiefly below it; farther towards the forehead gelatinous lymph only was found below the tendon, and serum only above it. The integuments at the side of the wound were sloughy. There was some little bleeding, stopped by pressure—a poultice was afterwards applied—she had salines with ammonia—and was allowed some red wine. In the evening the delirium was less violent—she was extremely low—and there was little secretion from the wounds.

On the 22d the prostration was yet more marked, and if the delirium was diminished it seemed only because her powers were sinking altogether. The induration of the face, particularly the right side, was extreme, and the tint of the erysipelas was almost cadaverous. Incisions were made in the face and eye-lids. In the latter there was gelatinous lymph mixed with pus, in the former gelatinous lymph only in the cellular texture. After the incisions she became so low as to require wine and egg, and brandy—towards evening she sank—and at midnight she died.

*Sectio Cadaveris.* There was great and universal effusion of lymph in some parts, pus in others, beneath the tendon of the occipito-frontalis. The cellular membrane was in many places sloughy, and separable with ease from the pericranium, as was the pericranium from the bone. At the original wound, the bone was bare for a little extent, and the superficial scale was beginning to turn brown and die.

There was a good deal of clear serous effusion between the membranes of the brain and in the ventricles, but otherwise the contents of the cranium were quite healthy. There was nothing unusual in the chest or abdomen.

In this case Mr. Brodie was prevented from seeing the patient with regularity in the early stages of the affection, and consequently the incisions were not made so promptly as in the preceding case. The perusal of the cases will shew the differences in the result.

In another report we shall take up the consideration of some further varieties of scalp-wound, and give cases illustrative of their secondary consequences.

---

XXI.

### RECTO-VESICAL LITHOTOMY.

### ST. GEORGE'S HOSPITAL.

### SCALP WOUNDS AND THEIR CONSEQUENCES.

IN our last report we detailed many cases of scalp-wound, and offered examples of the ordinary erysipelas of the scalp, and of the diffuse inflammation rapidly ending in sloughing, which frequently occurs in the cellular membrane beneath the tendon of the occipito-frontalis muscle.

There are certainly broad distinctions between these two affections, and those who have seen and watched both, will find little difficulty in recognizing either. But in this as in other things, it is difficult to draw the boundary line with absolute precision. In the diffuse inflammation of the cellular membrane beneath the occipito-frontalis there is often a degree of redness of the scalp, and even more is observed on its outskirts; whilst in the ordinary and genuine erysipelatous affection, there is more or less of swelling and œdema, from serous effusion above and below the tendon. For the diagnostic characters of each we refer to our last report; practical familiarity with cases will alone supply the nicer shades of difference that present themselves almost instinctively to the experienced surgeon.

The present report will be a short one. We shall mention the particulars of two cases exhibiting more curious and less explicable consequences of scalp wounds than those we have hitherto considered, and conclude by an example of the modes in which Nature repairs one portion of the cranium which has been denuded and deprived of its vitality.

CASE 1. *Lacerated Wound of the Scalp penetrating the Tendon of the Occipito-frontalis — Sloughing of the Cellular Membrane beneath that Tendon—Gangrene of the Right Lower Extremity.*

Isaac Sutton, æt. 53, admitted April 17, 1830, under the care of Mr. Babington.*

He was brought to the hospital at 5, p. m., with an ugly-looking, lacerated scalp-wound, situated over the right parietal bone, and not above an inch and half in extent any way; the bone was not denuded; there was sufficient bleeding to require several ligatures. The man was intoxicated and could give no account of himself whatever, but it was ascertained that he had been thrown from a cabriolet.

*Head shaved—poultice applied.*

On the next day he was still apparently intoxicated. There was no coma —the pupils were natural—there was no pain in the wound, but its edges were tumefied and its surface sloughy. He was ordered house physic, and the pulse rising in the evening he was *bled to* 12 ozs. On the 19th the report runs thus :—" pulse still too strong and rapid, much excitation."

*V. S. ad* ℥xij—*salines with antimony.*

On the 20th the pulse was nearly natural, but in the evening it was again becoming hard and rapid, and the V.S. was repeated to 12 ozs. with calomel and house physic. On the 21st the pulse was soft, but on the following day the pulse was quick and irritable, the tongue was furred, he had delirium, the scalp was tumid and œdematous, and there were some redness and swelling of the eye-lids.

---

* We only saw this patient on the day preceding his demise, and the details, which are taken from the book kept by the house-surgeon, are not so precise as could be wished.

*Mist. Camph. c. Ammon. carb.* gr. v. 6*tis horis.*

23d. The patient looks better, and has no pain in the head, but there is matter under the scalp. An incision down to the pericranium was made from the right ear to the vertex, and the cellular membrane beneath the tendon of the occipito-frontalis was found in a sloughing condition, the sloughs being mixed with a small quantity of pus. In the course of the day the patient complained of pain over the head of the right fibula, and an erythematous blush, about the size of a half-crown piece, with very slight tumefaction and no hardness, was observed there.

24th. Right upper eye-lid and side of face less swollen—pulse quiet—tongue white in the centre, clean and healthy at the edges—some delirium. A second incision, four inches long, was made in the course of the sagittal suture, and the sub-occipito-frontalis cellular membrane discovered in the same state as before. There was still pain in the leg with hardness and swelling of its whole outer side from the knee to the ankle.

The delirium increased in the night, and on the succeeding morning the right leg was in a state of mortification, with dark coloured vesications on its outer side, from the ankle to the middle of the thigh. The blood was coagulating, or at all events in a state of stasis, in the cutaneous veins. The patient sank rapidly and died at one, p. m.

*Sectio cadaveris* 24 *hor. post mortem.* The whole of the cellular membrane beneath the occipito-frontalis was in a sloughy state, and infiltrated with dirty-looking pus. The pericranium was very easily separable, but in no place actually separated from the bone. There was no fracture of the latter, nor any extravasation of blood above or below the dura mater. There was more serous fluid than usual between the arachnoid and pia mater, and likewise in the ventricles of the brain. The sinuses of the brain, as well as the jugulars and superficial veins of the neck, were healthy.

The heart was well proportioned, the aorta a little dilated at its end and thickened in its tunics. There was nothing particular in the abdomen. The right lower extremity, below the middle of the thigh, was in a state of mortification. The muscles and the other tissues were in a soft and semi-putrid state. The veins were perfectly free from inflammation.

CASE 2. *Scalp Wound denuding Bone to some extent—Rigors—Erysipelas of the Scalp and Face—Depôt in left Fore-Arm—sudden Death.*

James Talbot, æt. 39, admitted 1830, under the care of Mr. Brodie.

Scalp-wound an inch and a half in length, situated over the right side and upper part of the occipital bone, which is denuded to a small extent—small artery divided — much effusion of blood from the wound. Simple fracture of right clavicle. He fell down an area 10 feet deep upon his head, and was stunned at the time, but no sickness followed. *The wound dressed with adhesive plaster—figure of* 8 *bandage to the clavicle.*

On the 10th Sept. the wound was diminishing, but the bone was still exposed, and he complained of pain in the head and wound. In the night of the 4th the patient felt rather cold, and there being pain in the wound, an incision was made across it on the 13th, and the bone found exposed for a space the size of a shilling, but not dead. In the course of the day he had a rigor, without vomiting. On the 14th the pulse was 100. He was ordered salines. At 9, p. m. he had a rigor which lasted for a few minutes. Next morning he had nausea—pain in the head in the situation of the wound, which was rather flabby with white and smooth granulations—skin hot—pulse 132, weak—tongue white—pupils rather sluggish. *House physic.*

16th. Erysipelas, of palish yellow colour, has appeared upon the scalp, without much tumefaction—it extends over the forehead and down the back of the neck, where the tumefaction is more firm — wound glazed, dry, and pale—complains of frequent attacks of chilliness—aspect anxious—pulse 100, rather weak — tongue whitish — skin warm.

*H. sal. c. Vin. ant. t.* ℥ss. *4tis hor.*

On the next day the erysipelas had extended down the back of the neck and over the face with tumefaction and much tenderness, a dingy tint, and shining surface. The pulse was frequent and full, without force. *Ammoniated salines.* On the 18th the erysipelas had still extended, but the pulse was only 96, and there was less irritability. The scalp was still œdematous, but not red. He had no head symptoms — but on the 19th the erysipelas was spreading, and vesications had formed on the face, and produced scabs. There was much tumefaction of the right upper palpebra. The bowels were opened by senna. On the following day the erysipelas was spreading very little—there was and there had been much tumefaction—vesications on the nose filled with sero-purulent fluid. The œdema of the scalp was subsiding fast, leaving the scalp in wrinkles—the wound was pale but otherwise healthy, and granulations were forming on the bone. The pulse, &c., were nearly natural.

*Haust. cinchonæ ter die.*

On the 21st he was better, but some tympanitis was observed, and on the 23d this was increased, with uneasiness, referred chiefly to the right hypochondrium. The erysipelas was fading. Bowels not opened. *Vin. rub.* Oss.— *Pulv. rhei* gr. xv. — *Magnes. carb.* ℈i. *ex aq. menth. vir. statim, et rep.*

He vomited the draughts, the tympanitis was rather augmented in the evening, and a slight erythematous redness, with some tumefaction and much tenderness on pressure, were observed on the left fore-arm. *Cathartic enema —spirit lotion — draught of Magnes. sulph.*

23d. He is worse. Pulse 110, weaker —tongue becoming dry and red at the tip—bowels not freely opened. Desquamations on the face — some matter in the upper palpebræ. Nearly the whole of the outside of the fore-arm is now swollen, exquisitely painful on pressure, and suffused with a faint erythema. A puncture was made in the fore-arm and some matter discharged; the eye-lids were punctured with the same result. On the 24th the redness had extended over the elbow with a more defined margin. The belly was very tympanitic—the wound on the head was looking healthy—the general symptoms were improved. The bowels had never been freely opened, but this was readily effected by a large warm water injection. However the tympanitis was not a whit relieved, and on the 25th the patient was extremely low, the pulse being barely 60, and very irregular. Both the fore-arm and lower part of the arm were more swollen, but the redness on the latter was subsiding. Matter could be squeezed out from the puncture in the fore-arm. The tympanitis continued to distress the patient. On the 26th a cupping-glass was applied over the puncture, but no matter was extracted by that means. Another puncture was made in a suspicious part above the elbow, but only serum escaped. The erythematous redness was still present.

At midnight he died suddenly, having previously shewn no change for the worse. Unfortunately, owing to some blunder, an examination of the body was not obtained.

It may naturally be asked if the gangrene of the lower limb in the first case, and the affection of the fore-arm in the last, were more than accidental coincidences with the wounds of the scalp? Or it may be inquired if those remarkable occurrences were dependent, or not, upon the erysipelas? It is difficult to decide on points involved in so much obscurity; but, so far as our humble apprehension goes, we should be inclined to imagine, that the affections in question were more immediately connected with the erysipelas, than with the scalp wounds. Reasoning on such subjects is fallacious; the only useful guides are facts. Now it is not difficult to prove that purulent deposites in remote parts not unfrequently follow erysipelas, independent of any injury of the head, and mortification of the lower extremities occasionally occurs under similar, or nearly similar circumstances.

*Case.* Mr. Brodie amputated the breast of an old and unhealthy woman. Erysipelas followed on the wound. Suddenly the left lower extremity mortified, and the woman rapidly died.

*Case.* A man was lately in the hospital under Mr. Keate, with sloughing of the cellular membrane of the right thigh, in consequence of a severe contused wound. Suddenly the opposite limb, which had never been injured in any manner, mortified, and the man died.

These cases prove that mortification of the lower extremities may occur independent of injury of the head, whilst erysipelas or sloughing are going on in a distant part of the body. We need scarcely adduce cases to prove, that purulent deposites in remote parts are a frequent concomitant or consequence of erysipelas. All who have watched the practice of a large hospital must have witnessed many instances of this description. We may briefly mention two or three examples of the fact, which occur to our mind at the present moment. In August, 1828, a patient of Mr. Brodie's, affected with varicose veins and ulcers of the right leg, was attacked with a low kind of erysipelas of the limb. The erysipelas faded on the leg and thigh, but spread up the side of the body, typhoid symptoms and sinking rapidly came on, and he died on the fourth day. On dissection the right knee-joint was found filled with pus and its cartilages ulcerated, and there was sero-purulent fluid in the chest. A man was very lately in the hospital under the care of Mr. Keate, with erysipelas of the left leg, following a contused wound, and accompanied with some sloughing of the cellular membrane. The erysipelas was passing or had passed away, when a collection of matter formed in the opposite knee-joint and anterior part of the thigh. Small collections of matter appeared also on the fore-arms, and the patient died with the usual symptoms of the purulent deposites in the lungs. Owing to the decision of a Coroner's jury, no examination was obtained. A man pricked his thumb with a thorn. Inflammation and abscess followed and ulcerated openings led down to bare-bone. Seven weeks after the injury he was admitted into the hospital under Mr. Babington. In a week more, erysipelas followed and spread up the right arm and down the right side of the body. Tympanitis came on with typhoid symptoms, and the patient died on the 18th day. On dissection there was found matter in the right wrist-joint, and in the joints of the thumb. There was a small collection of matter near the left sterno-clavicular articulation; and in the left ankle-joint, where there had been a puffy painful swelling during life, with erythema, there was found a mixture of pus and synovia, with inflammation of the neighbouring periosteum and cellular membrane. We might instance many more cases of this kind, but enough has been said to place the fact, of collections of matter occurring in distant parts as a consequence or concomitant of erysipelas, beyond all question or dispute.

From these considerations we are induced to believe that the gangrene of the lower extremity, in the case of Sutton, and the collection of pus in the fore-arm, in the case of Talbot, were rather dependent on the erysipelatous affections than on the scalp-wounds or injuries of the head, *per se*. At the same time we advance the suggestion with diffidence, for our knowledge on these subjects is at present very imperfect. Something too may be said on the other side of the question. It has been observed since the days of Dessault, that injury of the head is frequently followed by purulent deposites in the liver. Mr. Brodie, to whose practical knowledge and instructions we are proud to say that we owe all, has remarked that after injuries of the head slight wounds will run into suppuration, and matter is apt to form about simple fractures. There was an insance of this the other day at the hospital. A patient of Mr. Babington's had compound fracture of the skull. He was trephined and all did well about the head. But he had also a simple fracture of the thigh. Here a large collection of matter took place, hectic was established, amputation was performed as a last and desperate chance, and the patient died from the shock of the operation. On dissection there was some recent inflammation of the lungs, unquestionably that "peripneumony of the dying" described by Laennec.

These considerations must give us pause before we pronounce a dogmatical opinion on subjects involved in so much obscurity. But still we think the probabilities are in favour of the affections of the extremities in the cases of Sutton and Talbot, having rather depended on the erysipelas than on the injury of the head. The erysipelas itself was the consequence of the scalp-wounds, and therefore the cases are properly and naturally to be considered along with the other injuries of the head.

We shall now relate a case exemplifying the method by which the outer table, or a part of the outer table, of the cranium is removed, when deprived of vitality by local injuries.

CASE 3. *Extensive lacerated Scalp-Wound—Bone denuded—Cure.*

William Maples, a servant, æt. 42, admitted June 4, 1830, under Mr. Hawkins.

Very severe and extensive lacerated scalp-wound, occupying the top and the right side of the head—part of the scalp torn away — the remainder hanging in flaps, with much dirt and gravel, and considerable effusion of blood around. Bone denuded for a space as large as a shilling. A good deal of bleeding. The accident happened by his falling from behind a carriage in Grosvenor Place. He was not stunned, but felt rather sick. He had drunk freely.

*Wound well cleaned—lint soaked in blood applied with adhesive straps—cold lotion.*

Bleeding subsequently took place to some little extent, and two days afterwards, when suppuration had commenced, a quantity of thin bloody fluid ran out from the wound. There was little fever. A good deal of ecchymosis about the eye took place, and on the 19th coagula of blood were discharged from the nose. A slight chill had occurred on the preceding day. The treatment consisted of salines with mag. sulph. and vin. ant. The wound was carefully dressed every day. No unfavourable symptoms whatever occurred, and about the middle of July he was made an out-patient.

The edges of the wound had granulated well, and the wound itself was contracted to about the diameter of a crown-piece. A superficial layer of the outer table of the bone had died. In parts it was absorbed, and through numerous little apertures thus formed, granulations had sprung up. The edges too of the dead scale of bone were loosened and separated from the bone in the neighbourhood, by a kind of trench, formed by ulcerative absorption. Thus the process adopted by nature to get rid of the effete and useless layer of bone, was in part exfoliation, in part ulceration or absorption.

In another case a nearly similar process was observed. A large surface of bone was exposed by sloughing of the cellular membrane under the occipito-frontalis. Its dimensions were gradually diminished by granulations hemming it in on all sides. Then red spots like little ecchymoses checkered the yellow-white surface of bone. These increased in size, and after a time a granulation peeped through a minute opening. At last the whole surface granulated in this manner, and no perceptible exfoliation took place. Particle after particle of the dead bone appeared to have been removed gradatim by absorption, and granulations filled its place.

---

